# Fabry Disease in a Female: A Unique Case Highlighting the Variability in Clinical Presentation

**DOI:** 10.7759/cureus.70406

**Published:** 2024-09-28

**Authors:** Arshiya Gupta, Sumedh R Luthra, Shivansh Luthra

**Affiliations:** 1 Department of Medicine, Government Medical College Amritsar, Amritsar, IND

**Keywords:** alpha-galactosidase a deficiency, cardiovascular complications, enzyme replacement therapy, fabry disease, genetic mutations, heterozygous female, multisystem involvement, neurological symptoms, renal dysfunction, x-linked disorder

## Abstract

Fabry disease is a rare X-linked lysosomal storage disorder caused by mutations in the GLA gene, leading to deficient activity of the enzyme alpha-galactosidase A. This enzyme deficiency results in the accumulation of globotriaosylceramide (Gb3) in various tissues, causing multi-systemic manifestations. This case report presents a rare instance of Fabry disease in a 32-year-old female patient, highlighting the unique clinical presentation with multisystem involvement. Fabry disease is an X-linked lysosomal storage disorder that primarily affects males, while females are often considered asymptomatic carriers. Heterozygous females can exhibit a broad spectrum of clinical manifestations, varying from a complete absence of symptoms to the full expression of the disease. The patient presented with a complex array of symptoms, including progressive dyspnea, fever, headache, lower limb pain, and periorbital edema, accompanied by a history of hypertension and chronic kidney disease (CKD). Laboratory investigations revealed severe anemia, elevated renal function parameters, and significant proteinuria. A renal biopsy confirmed the diagnosis of Fabry disease, based on the characteristic histopathological findings of widespread glomerular and segmental tuft sclerosis, as well as podocyte enlargement with fine vacuolization. The patient was managed with a combination of sequential hemodialysis and diuretic therapy. This case is a rare and unique example of Fabry disease in a female patient, with symptoms affecting multiple organ systems, including the renal, cardiovascular, and neurological systems. It underscores the importance of maintaining a high index of suspicion for Fabry disease, even in female patients, and the need for a comprehensive diagnostic approach to ensure timely diagnosis and appropriate management. Early recognition of this rare condition in females is crucial for the implementation of targeted therapies, such as enzyme replacement therapy and oral chaperone therapy, to prevent the progression of multi-organ damage.

## Introduction

Fabry disease, an X-linked lysosomal storage disorder, arises from a deficiency or absence of alpha-galactosidase A (α-GAL A) activity. This enzymatic dysfunction is attributed to genetic mutations in the GLA (galactosidase alpha) gene, located at Xq21.3-q22, resulting in the lysosomal accumulation of glycosphingolipids, predominantly cerebroside trihexoside [1]. Consequently, affected individuals exhibit a significantly elevated risk of developing ischaemic stroke, small-fiber peripheral neuropathy, cardiac dysfunction, and chronic kidney disease (CKD) [2]. The estimated prevalence of Fabry disease is approximately one in 40,000 individuals [3]. However, newborn screening suggests a higher incidence of 1:3, 100 to 1:1500 male births [3,4]. The disease affects all ethnic groups, with males more severely affected than females [5].

Historically, females were considered to be asymptomatic carriers of defective GLA genes who were not vulnerable to the symptoms and vital organ abnormalities [6]. However, recent research has revealed that heterozygous females can exhibit a broad spectrum of clinical presentations, ranging from the complete absence of symptoms to a full manifestation of the classic disease [6]. This variability in disease expression may be attributed, at least partially, to skewed X-chromosome inactivation [7]. This process could lead to a higher proportion of cells expressing the disease-causing mutation in affected organs, resulting in more severe clinical outcomes. Hypertension is frequently observed in Fabry disease patients, primarily due to progressive renal impairment [4]. Both diagnosis and management of Fabry disease present considerable challenges as the disease presents with a broad spectrum of clinical manifestations, ranging from early-onset, severe forms to later-onset variants primarily affecting the heart and kidneys. Early diagnosis and enzyme replacement therapy (ERT) or chaperone therapy are crucial in mitigating organ damage and improving long-term outcomes. However, when organ-specific symptoms are evaluated in isolation, the underlying systemic etiology may be overlooked [5,6].

We present a rare case of Fabry disease in a female patient, exemplifying the intricate interplay between molecular pathology and multisystem clinical manifestations. The resultant deficiency or dysfunction of alpha-galactosidase A in this patient leads to the progressive accumulation of globotriaosylceramide within lysosomes, primarily affecting the vascular endothelium, kidneys, myocardium, and nervous system.

## Case presentation

A 32-year-old female with a known history of hypertension and CKD presented to the emergency department with a complex array of symptoms. The patient's chief complaints included progressive dyspnea over a 10-day period, low-grade to moderate fever (100°F to 102°F) for four days, and a three-month history of headache, lower limb pain, and periorbital edema. Her past medical history was significant for oliguria accompanied by frothy urine and orthopnea. The patient had previously undergone three sessions of hemodialysis and had received blood transfusions. Obstetric history revealed the patient to be P3A1L2 (three pregnancies, one abortion, and two living children).

Upon physical examination, the patient was found to be in a stable general condition, albeit with notable vital sign abnormalities. Her blood pressure was elevated at 170/90 mmHg, with tachycardia (110 beats per minute) and tachypnea (35 breaths per minute). The patient was febrile (101°F) with an oxygen saturation of 94% on room air. Random blood glucose was 137 mg/dL. Clinical examination revealed pallor and edema, but no icterus, clubbing, cyanosis, or lymphadenopathy. Cardiovascular and gastrointestinal examinations were unremarkable. Respiratory auscultation demonstrated bilateral basal crepitations. Neurologically, the patient was fully conscious with a Glasgow Coma Scale score of 15, although she reported paresthesia. Laboratory investigations revealed significant hematological and biochemical abnormalities (Table 1).

**Table 1:** Summary of the patient's laboratory findings along with the normal ranges

Parameter	Patient’s values	Normal ranges
Random blood glucose	137 mg/dL	70-140 mg/dL
Hemoglobin	5.2 g/dL	12-16 g/dL (female)
Total leukocyte count	7100/mm³	4000-11000/mm³
Blood urea	109 mg/dL	7-20 mg/dL
Serum creatinine	5.6 mg/dL	0.6-1.2 mg/dL
Sodium	137 mEq/L	135-145 mEq/L
Potassium	4.8 mEq/L	3.5-5.0 mEq/L
Prothrombin time index (PTI)	85%	70-100%
Proteinurea	+++	Negative

The patient's hemoglobin was markedly reduced at 5.2 g/dL, indicative of severe anemia. While the total leukocyte count (7100/mm³) and differential counts were within normal ranges, renal function tests were notably abnormal. Blood urea (109 mg/dL) and serum creatinine levels were substantially elevated (5.6 mg/dL), confirming significant renal impairment. Electrolyte analysis showed normal sodium (137 mEq/L) and slightly elevated potassium (4.8 mEq/L) levels. Liver function tests and coagulation parameters (PTI 85%) were within normal limits, and viral markers were non-reactive. Urinalysis demonstrated significant proteinuria (+++) without glycosuria, ketonuria, or pyuria. Renal ultrasonography suggested bilateral medullary disease.

**Figure 1:**
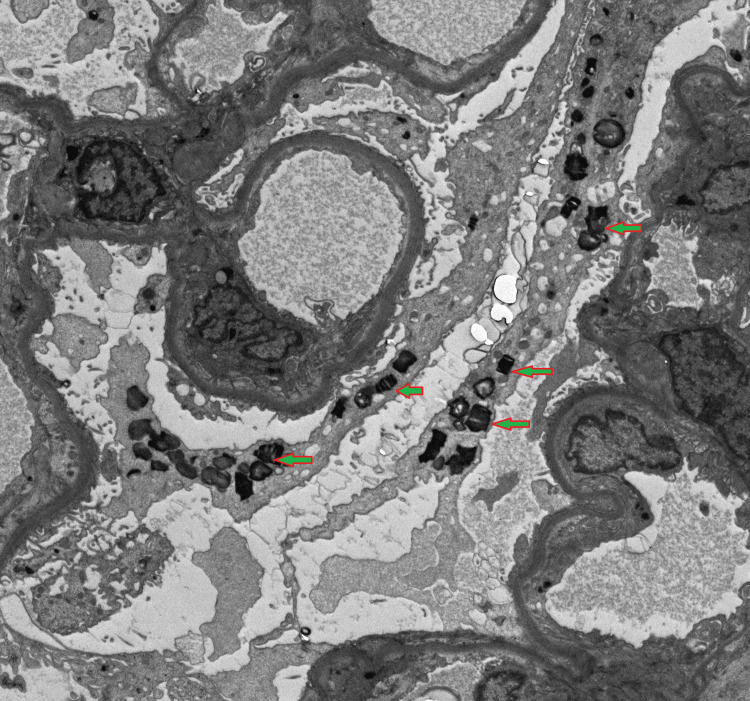
Proband first biopsy images under electron microscopy revealing numerous lamellated inclusion Zebra bodies in the glomerular visceral and tubular epithelial cells confirming Fabry disease.

**Figure 2:**
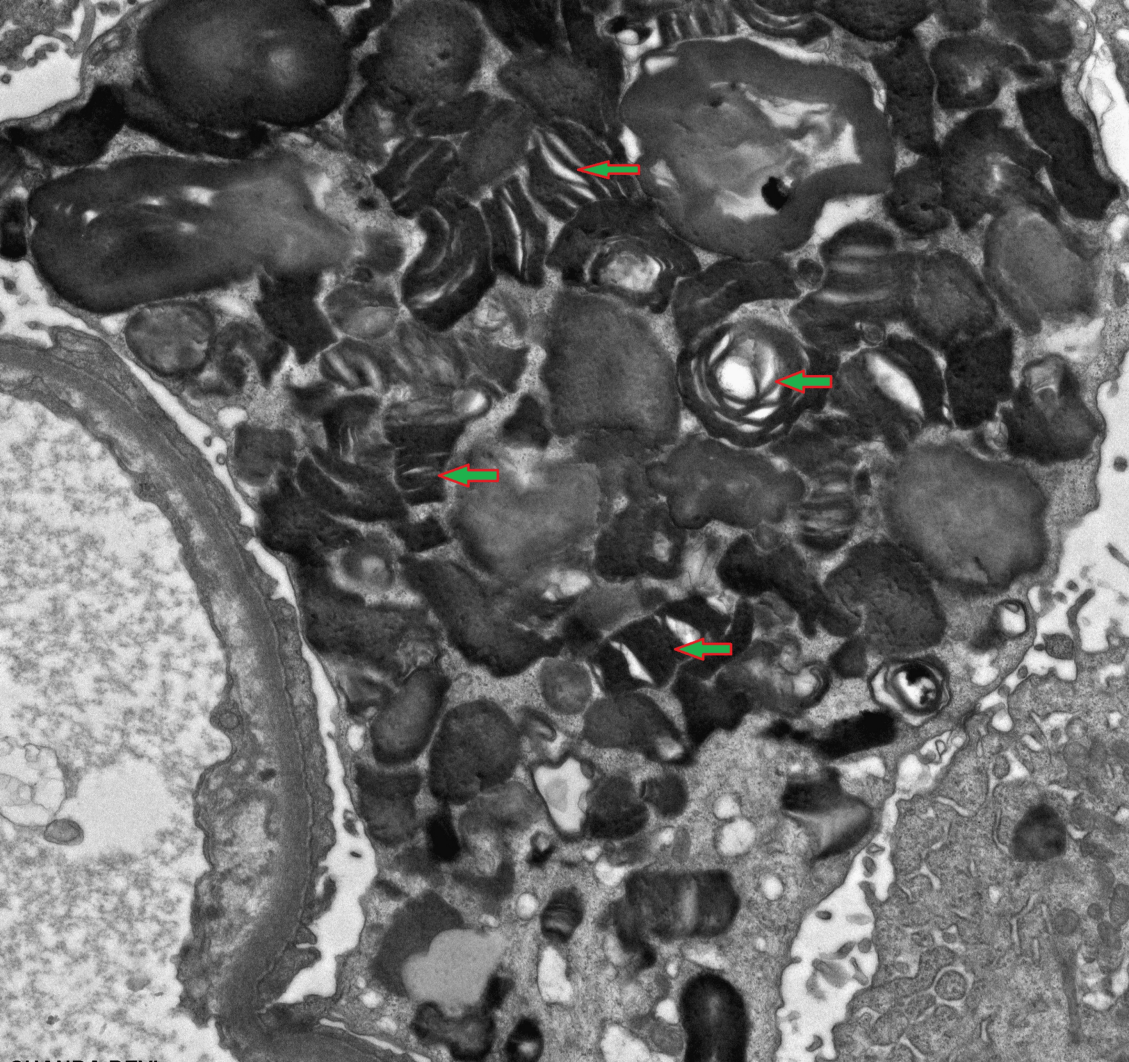
Renal electron microscopy images taken from the probands' second biopsy. Zebra bodies are still present.

A renal biopsy was considered necessary because the patient had significant renal impairment, including CKD, elevated renal function parameters (blood urea and serum creatinine), and marked proteinuria, which prompted further investigation to elucidate the underlying pathology (Figures 1, 2).

**Figure 3:**
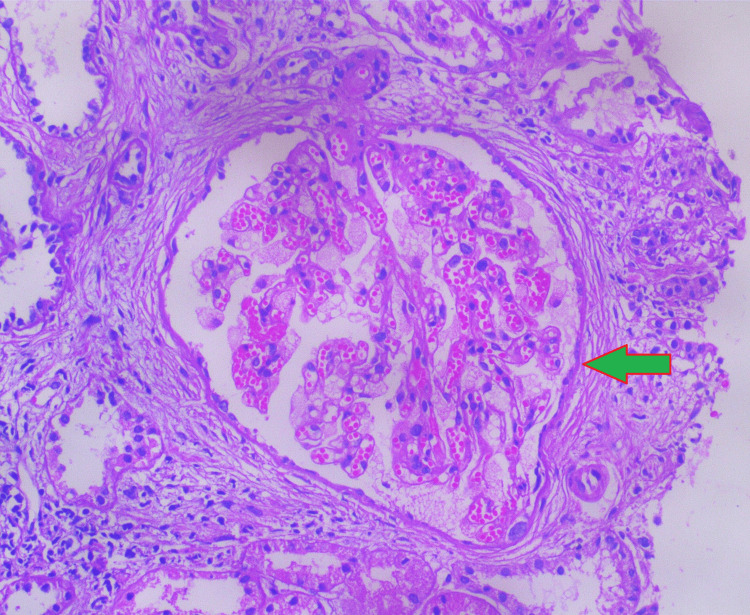
Histopathological findings confirming diffuse fine vacuolization and remarkable cytoplasmic expansion of visceral epithelial cells/podocytes (stained with H&E, periodic acid Schiff (PAS), silver methenamine, Masson's trichrome (MT), and Congo red)

**Figure 4:**
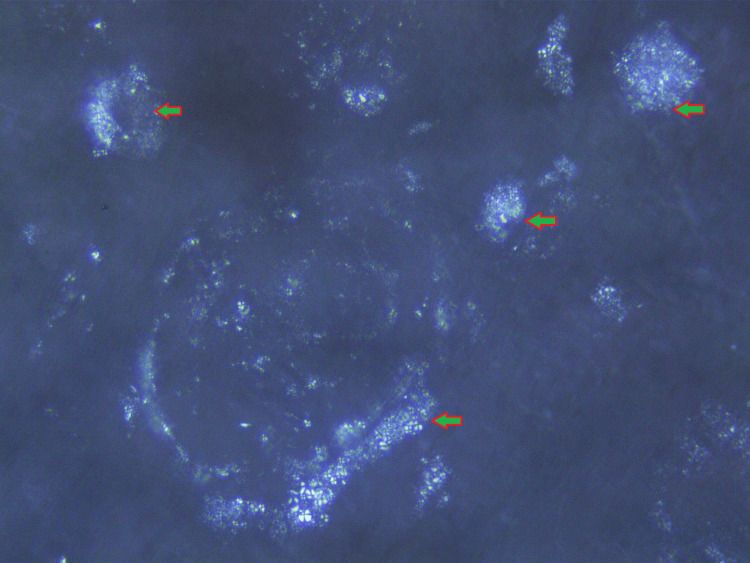
Direct immunofluorescence image showing renal medulla and cortical parenchymal area containing many globally sclerosed glomeruli.

Histopathological examination revealed widespread glomerular and segmental tuft sclerosis within the renal medulla and cortical parenchyma. Notably, the podocytes exhibited enlargement with fine vacuolization, a characteristic finding in Fabry disease (Figures 3, 4).

However, no evidence of crescent formation, tuft necrosis, intracapillary thrombi, or congophilic deposits in the visualized glomeruli was noted. These histological features, combined with the clinical presentation and biochemical profile, led to a definitive diagnosis of Fabry disease. The patient's management involved sequential hemodialysis and diuretic therapy. To control hypertension, the patient was started on enalapril at a dose of 5 mg twice daily, with a target range of 5-40 mg per day. Follow-up was scheduled every four to six weeks to monitor blood pressure, renal function (estimated glomerular filtration rate (eGFR), creatinine), and proteinuria. The patient was ultimately discharged in satisfactory condition on a regimen of maintenance hemodialysis.

## Discussion

In females, the onset of Fabry disease symptoms mirrors that in males, but with a lower prevalence at any given age [7]. Heterozygous females can experience a range of symptoms, including acroparesthesias, angiokeratomas, hypohidrosis, cornea verticillata, chronic abdominal pain, and diarrhea [8]. With age, they may develop cardiac issues (fibrosis, left ventricular hypertrophy (LVH), arrhythmias, valvular disease), cerebrovascular events (transient ischemic attacks (TIAs), strokes), and, less frequently, kidney problems (proteinuria, CKD) [8]. When CKD occurs, the progression to end-stage kidney disease is similar in males [9]. Cardiac, cerebrovascular, and kidney diseases generally appear a decade later than in males [9]. A Fabry Registry study of 1,077 females found that 70% reported symptoms, with a median onset age of 13 years, and 20% experienced major events by a median age of 46 years [10].

The present rare case of lysosomal storage disorder explains the multisystemic manifestations observed in the female patient, including renal dysfunction, hypertension, and neurological symptoms. Current guidelines recommend screening for Fabry disease in patients exhibiting at least two of the following clinical features: reduced sweating (anhidrosis or hypohidrosis), angiokeratomas (a characteristic reddish-purple skin rash localized to the bathing trunk area), or a personal and/or family history of renal failure [11]. Furthermore, a history of acroparesthesias, characterized by burning or hot pain in the extremities, particularly during febrile episodes, or a history of exercise, heat, or cold intolerance, should prompt further investigation [12]. Diagnostic evaluation is also warranted in individuals with sporadic or non-autosomal dominant patterns of unexplained cardiac hypertrophy, especially in the absence of male-to-male transmission [13].

Microscopic urinalysis can reveal mulberry bodies in the sediments, which are distal epithelial cells with accumulated globotriaosylceramide exhibiting a characteristic whorl-like appearance [14]. Renal biopsy may demonstrate the Maltese cross pattern due to the birefringence of lipid droplets, primarily consisting of cholesterol esters [15]. Treatment strategies for Fabry disease encompass a multifaceted approach, including anticoagulation and enzyme replacement therapies, such as agalsidase beta and pegunigalsidase alfa [16]. Recent advancements have introduced oral chaperone therapy (migalastat) as an additional treatment option. Patient management involves sequential hemodialysis and diuretic therapy [17-18]. This case underscores the importance of a high index of suspicion for Fabry disease in patients presenting with multisystem involvement, particularly renal dysfunction, to ensure timely diagnosis and appropriate management.

## Conclusions

The present case illustrates the significance of recognizing Fabry disease in female patients, highlighting the need for heightened clinical awareness. The patient's presentation with multisystem involvement, including renal dysfunction and neurological symptoms, underscores the complexity of diagnosing Fabry disease in females who can exhibit a wide spectrum of clinical manifestations. Early diagnosis is crucial to initiate appropriate treatments, such as enzyme replacement and oral chaperone therapies, to mitigate disease progression and improve patient outcomes. This case emphasizes the importance of a comprehensive approach to diagnosing and managing Fabry disease, even in populations traditionally considered at lower risk.
